# Echo Chambers and Homophily in the Diffusion of Risk Information on Social Media: The Case of Genetically Modified Organisms (GMOs)

**DOI:** 10.3390/e27070699

**Published:** 2025-06-29

**Authors:** Xiaoxiao Cheng, Jianbin Jin

**Affiliations:** 1 College of Media and International Culture, Zhejiang University, Hangzhou 310058, China; 2 School of Journalism and Communication, Tsinghua University, Beijing 100084, China; jinjb@tsinghua.edu.cn

**Keywords:** Shannon entropy, echo chamber, homophily, risk, information sharing, GMOs, social contagion theory

## Abstract

This study investigates the mechanisms underlying the diffusion of risk information about genetically modified organisms (GMOs) on the Chinese social media platform Weibo. Drawing upon social contagion theory, we examine how endogenous and exogenous mechanisms shape users’ information-sharing behaviors. An analysis of 388,722 reposts from 2444 original GMO risk-related texts enabled the construction of a comprehensive sharing network, with computational text-mining techniques employed to detect users’ attitudes toward GMOs. To bridge the gap between descriptive and inferential network analysis, we employ a Shannon entropy-based approach to quantify the uncertainty and concentration of attitudinal differences and similarities among sharing and non-sharing dyads, providing an information-theoretic foundation for understanding positional and differential homophily. The entropy-based analysis reveals that information-sharing ties are characterized by lower entropy in attitude differences, indicating greater attitudinal alignment among sharing users, especially among GMO opponents. Building on these findings, the Exponential Random Graph Model (ERGM) further demonstrates that both endogenous network mechanisms (reciprocity, preferential attachment, and triadic closure) and positional homophily influence GMO risk information sharing and dissemination. A key finding is the presence of a differential homophily effect, where GMO opponents exhibit stronger homophilic tendencies than non-opponents. Despite the prevalence of homophily, this paper uncovers substantial cross-attitude interactions, challenging simplistic notions of echo chambers in GMO risk communication. By integrating entropy and ERGM analyses, this study advances a more nuanced, information-theoretic understanding of how digital platforms mediate public perceptions and debates surrounding controversial socio-scientific issues, offering valuable implications for developing effective risk communication strategies in increasingly polarized online spaces.

## 1. Introduction

The proliferation of social media has fundamentally changed the landscape of science and risk communication, transforming how technological risk-related information is disseminated, shared, and collectively interpreted and perceived by the public. Unlike the “one-to-many” broadcasting diffusion pattern, social media platforms facilitate a “many-to-many” paradigm of information dissemination, enabling information to spread across complex, decentralized networks [1]. This transformation is underpinned by the social-relational architecture of digital platforms, which allows users to establish weak ties with minimal effort through behaviors such as one-click sharing. These affordances substantially lower barriers to information dissemination and amplify the potential for behavioral social contagion [2], thereby triggering large-scale information cascades [3] that make risk messages “go viral.”

Information diffusion encompasses two distinct yet interrelated processes crucial to understanding how risk information spreads through digital networks. The first is the dissemination of the information itself [4,5], referring to how facts, opinions, and narratives about risk topics propagate across networks—essentially focusing on what messages are being spread, how they are framed, and how they are processed by users. The second is the transmission and contagion of behavioral decisions and actions [2,6,7], which pertains to how individuals’ choices to engage with content (e.g., sharing and reposting) spread through social networks as users observe and potentially mimic others’ behaviors. As Liang and Kee [8] define it, information diffusion encompasses “the extent to which individuals engage in communication behaviors that expose themselves to, interact with, and/or pass on online content” (p. 4), highlighting both message content and user behaviors as fundamental mechanisms.

While these processes are interconnected, behavioral transmission deserves independent analytical attention because sharing decisions may be influenced by factors beyond information content itself. Users might share content not because they find it particularly compelling but because they observe others in their network doing so, or because sharing aligns with their social identity and group membership. From a cognitive neobehaviorist perspective, these sharing behaviors can be understood as learned responses that are mediated by cognitive processes, where environmental stimuli interact with internal cognitive structures to produce behavioral outcomes [9,10]. Personal motivations, social context, network position, and the desire to maintain social relationships all play crucial roles. This complexity is particularly important because social media users’ engagement in information diffusion represents an individual behavioral decision made after cognitive and heuristic processing [7], which is pivotal not only for widespread information propagation but also for triggering cascading behaviors through networked public participation.

Despite increasing academic attention to content-based features that trigger message dissemination (e.g., references [11,12,13]), the behavioral aspect of information diffusion remains underexplored. Existing research has primarily focused on identifying which content characteristics make messages “sharable” while paying insufficient attention to the social mechanisms driving individual sharing decisions. Information sharing generates more cross-user interactions than viewing and commenting activities [14], partly because sharing behavior has been imbued with meaning and significance. It involves a high degree of behavioral commitment [15], which confers upon the shared information certain qualities of social acknowledgment and endorsement [16]. Consequently, information flow enabled by the social contagion of sharing decisions and actions—rather than information itself—functions as a significant factor that reduces the cost and enables effective and widespread mobilization [16,17] in public engagement with communal risk issues.

From an information-theoretic perspective, the diffusion of information on social media—especially regarding risk topics—resembles a process of uncertainty reduction through information exchange within a networked system. The behavioral dimension can be conceptualized as an influence cascade, where actions propagate through chains of users, each influenced by external stimuli or intrinsic motivations, who then influence others. Shannon’s entropy provides a mathematical framework for quantifying uncertainty in information transmission and reception, effectively capturing the degree of disorder, diversity, and predictability in information flow patterns. This makes entropy-based methods particularly valuable for analyzing risk communication dynamics where uncertainty is inherent, allowing us to quantify the uncertainty and concentration of attitudinal differences and similarities among users and providing a more nuanced understanding of how social factors influence behavioral transmission beyond content characteristics alone.

The context of genetically modified organisms (GMOs) on Weibo, a Chinese Twitter-like social media platform, offers an ideal empirical setting for examining the behavioral aspect of risk information diffusion. GMOs represent a highly contentious socio-scientific issue in China and beyond [18,19,20], marked by polarized public attitudes, scientific uncertainty, and significant policy implications [21,22]. The diffusion of GMO-related risk information on social media not only illuminates the mechanisms of information spread but also provides critical insights into broader patterns of public engagement with controversial socio-scientific topics. In hybrid digital environments where misinformation and polarization are prevalent, a deeper understanding of these behavioral dynamics addresses a significant gap in the diffusion literature, where the focus has often been on the content of information. Such understanding is essential for developing effective risk communication and governance strategies, particularly those that leverage behavioral interventions to enhance public engagement and response to risk-related issues.

Therefore, the present study seeks to identify the mechanisms that drive the social sharing of GMO risk information on Weibo, with a particular emphasis on the dyadic relationships formed through individual sharing behavior and the role of entropy in characterizing attitudinal alignment/similarity and uncertainty. Drawing on social contagion theory [2], we investigate the roles of both exogenous and endogenous mechanisms—specifically, homophily and network structural forces including preferential attachment, reciprocity, and triadic closure—in shaping information sharing patterns within the context of GMO risk communication. By employing both Shannon entropy-based analysis and Exponential Random Graph Model (ERGM), this study disentangles the complex social interactions that influence user engagement in sharing behaviors amongst users with diverse attitudes toward GMOs.

## 2. Literature Review

### 2.1. Risk Communication and Social Contagion Theory

Risk is both fact-laden and value-laden, manifesting a synthesis of objective reality and subjective perception [23,24]. People construct and shape social risks through interactions and communication based on risk information. In this regard, social contagion of risk explains the very logic of how social risk perceptions form and risk messages spread, particularly regarding controversial socio-scientific issues like GMOs.

Traditional approaches to studying risk perception and communication have predominantly focused on individual-level analyses, examining cognitive mechanisms while treating risk-related perceptions, decisions, and behaviors as atomized units disconnected from broader social systems [25,26]. Such individualistic approaches fail to capture the complex social dynamics and contagion mechanisms that shape how risk information spreads through networks and how collective risk perceptions emerge. As noted by Scherer and Cho [26], individual-level theories cannot adequately explain variations in risk perception between groups or within community clusters. This limitation becomes especially problematic when the interdependence between individuals facing risk increases, requiring them to balance conflicts of interest between themselves and other members of the collective [27]. These shortcomings call for a network-based perspective that can better account for social influences as well as constraints on risk information sharing and diffusion.

Social networks nurture and foster human behaviors, and the corresponding behavior cascades as people’s attitudes, decisions, and actions affect and are influenced by those of their peers [28]. Such behavioral interdependence across social networks has been observed in various domains [29], and it is widely accepted that online social messages conveying attitudes and behaviors are highly susceptible to social contagion effects [30]. Social contagion refers to the process through which individuals adopt behaviors or attitudes from others via observational learning within their social networks [2,31,32]. This phenomenon emphasizes how the structural forces of one’s embedded social network can produce significant attitudinal and behavioral contagions as well as convergence [28,29,33], making it particularly relevant for understanding the behavioral aspect of risk information diffusion in digital media environments.

Building on this network perspective, Scherer and Cho [26] proposed the “social network contagion theory of risk perception,” drawing upon Burt’s [31] “network theory of contagion.” This theory posits that risk perception and communication are shaped not merely by isolated individual cognitive processes but by the relational aspects of individuals and the resulting networks, which resemble self-organizing systems. This theoretical advancement represents a significant shift toward understanding risk as a socially constructed phenomenon that emerges from network interactions.

Recent studies on information sharing have further highlighted the impact of “interdependence” among users in risk information diffusion and behavioral decisions [3,27]. These studies incorporate theoretical models such as the theory of reasoned action [34] to examine how factors like perceived norms, social influence, perceived community cohesiveness, and belief in reciprocity influence risk information sharing behaviors [27,35]. By integrating social influence factors into models of risk sharing, these approaches, to some extent, bridge the gap between individual and network perspectives.

Nevertheless, research on the social contagion of risk still faces significant methodological limitations [33]. Specifically, prior studies rely heavily on survey questionnaires to measure users’ perceptions as proxies for social network influence, rather than analyzing actual network structures and behaviors. Also, when measuring risk information sharing, researchers often focus on behavioral intentions rather than actual sharing behaviors. Even when studies attempt to measure social network influence based on relational data—such as Scherer and Cho [26], who measured the frequency of daily communication among community residents—they typically rely on self-reported nomination methods that are susceptible to omitted ties and biased estimations [33].

These methodological challenges highlight the need for an approach that can capture actual sharing behaviors and network structures, particularly in the context of social media platforms where digital behavioral data is traceable. In the following section, we employ a Multi-Theoretical Multilevel model (MTML) [36] that enables simultaneous testing of different tie-generative mechanisms underlying the formation of GMO risk information-sharing networks. This approach allows us to better understand how network structural forces and homophily mechanisms influence the diffusion of risk information.

### 2.2. Generative Processes Driving Information Sharing

Investigating the behavioral dimension of online information transmission from a network-based social contagion perspective essentially explores tie-generative mechanisms of information sharing networks among users. The MTML framework offers a useful overarching analytical framework to understand the mechanisms that drive the formation of GMO risk information-sharing networks. Prior studies have employed the MTML framework to understand the creation, maintenance, dissolution, and reconstruction logics [36] of online interactions and the assembly of self-organizing teams in virtual communities [37,38].

According to MTML, tie formation cannot be attributed to a single theory or analytical level; instead, it requires the juxtaposition of multiple theoretical mechanisms across different levels of analysis [37]—such as individual, dyadic, and triadic levels [39]—to clarify the influence of each mechanism. Previous studies have largely examined and confirmed two sets of tie-generative mechanisms driving online social interactions: endogenous network structural forces and exogenous individual attributes [37,40,41,42,43].

#### 2.2.1. Endogenous Mechanisms

The endogenous mechanisms address how the presence of some ties encourages others to come into existence [38], reflecting the inherent interdependence of network relationships [37]. This line of research examines how relationship formation between individuals is influenced by the network structure in which users are embedded [30,44,45].

Three basic types of structural forces have been widely accepted that could generate network self-organization: preferential attachment, reciprocity, and triadic closure [37,40,42,43,46,47]. The preferential attachment mechanism, based on the scale-free network model proposed by Barabási and Albert [46], assumes that new members join a continuously growing social network one by one. When new nodes join, they are more likely to connect with nodes that already have a higher degree [48]. Networks formed under preferential attachment exhibit a power-law distribution of node degrees, where a small number of nodes have extremely high degrees, while most nodes reside in the long tail [42,46,47,49]. Researchers commonly conceptualize this mechanism as “popularity” [15,43] or “prestige” [42] when measuring its effect on tie formation.

Reciprocity and triadic closure represent two additional endogenous mechanisms for online interactions; both originate from Heider’s balance theory [50], which suggests that humans naturally seek symmetry in social relationships [40]. However, these mechanisms differ in their focus and analytical level. The norm of reciprocity, a fundamental principle in human society, suggests that people tend to return favors [38,40]. In networks with stronger reciprocity, the mutuality of relationships between nodes is higher—meaning any pair of nodes is more likely to “choose” each other and become adjacent. While most studies confirm the universality of reciprocity in establishing network relationships, asymmetrical ties (non-reciprocity) also play important roles. For example, Peng et al. [40] found that both reciprocity (mutual following on Twitter) and asymmetrical ties (one-way following) among US congressional members increased the probability of interaction in their Twitter mention network.

Unlike reciprocity, which operates at the dyadic level, triadic closure or transitivity operates at the triadic level, where two users connected to a third person also connect with each other—essentially, “friends of friends become friends” [37,38,39,40]. In triadic closure, each node serves as an intermediary for the other two nodes, thereby driving the formation of a more cohesive network structure.

Based on the review of endogenous mechanisms, we propose the following research question:

**RQ1:**  Do endogenous network mechanisms (preferential attachment, reciprocity, and triadic closure) influence GMO risk information-sharing behavior and, if so, how?

#### 2.2.2. Homophily Mechanism

Beyond the aforementioned endogenous structural mechanisms, users also tend to form connections with others who possess similar exogenous attributes—a phenomenon known as homophily [45,51,52]. Empirical studies typically conceptualize homophily as user similarity across multiple dimensions, broadly categorized as baseline homophily and inbreeding homophily [37,53]. Baseline homophily refers to relationship formation based on similar actor attributes such as age, gender, and geographical location; inbreeding homophily focuses on how shared institutional affiliations influence network interaction relationships, also known as “institutional homophily” [54]. In this line of research, studies have consistently confirmed the prevalence of homophily effects across various contexts, including political communication [40,55,56], health communication [37,38,43], environmental communication [42,57,58], and other fields [59].

In the context of GMO risk information diffusion, this study focuses on positional homophily, which exists at the intersection of baseline and institutional homophily. Positional homophily can be viewed as the tendency of individuals to form relationships with others who share similar attitudinal positions or viewpoints [42,60] on GMO risks. This type of homophily is particularly relevant in the GMO context because public attitudes toward GMOs function both as individual actor attributes and as potential catalysts for social identity formation. This dual nature of GMO attitudes makes positional homophily characteristic of both baseline and institutional homophily, making it a crucial mechanism for understanding how GMO risk information spreads online.

A theoretical question worth clarifying and further exploring: what substantive impact will positional homophily have on the online public sphere? Although homophily can strengthen connections among like-minded peers and further facilitate the generation of social networks, an increasing number of studies recognize that positional homophily is likely to trigger selective sharing behavior [61,62] and generate the “echo chamber effect” [47,63,64,65,66,67], ultimately leading to the disintegration or separation of social networks.

However, some recent studies have challenged this conclusion [68]. For instance, one recent study directly refutes the echo chamber effect based on discussions of the 2016 US election on Reddit [66]. It was found that cross-cutting political interactions between two different user groups with opposing political stances (Trump supporter or Hillary supporter) were significantly higher than expected, thus confirming a “heterophily” [69] rather than a homophily mechanism.

Regarding the GMO issue, similar debates exist. Most studies have demonstrated that individuals tend to gravitate toward like-minded groups, adhering to their own views and setting their own communication agendas, which forms distinct camps with clear boundaries between opposing viewpoints (“pro-GMO” and “anti-GMO”) [22] and makes effective dialogue difficult to achieve [21]. However, a more recent study offers completely opposite evidence [67] that GMO supporters and opponents engage in a significant number of cross-stance interactions (commenting behavior).

Based on these inconsistent findings regarding positional homophily, we propose the following research question:

**RQ2:**  Does the positional homophily mechanism influence GMO risk information-sharing behavior, and if so, how?

Notably, an emerging stream of research has increasingly paid attention to intergroup differences in homophily effects, identifying two distinct forms: uniform homophily and differential homophily [38,70]. Specifically, uniform homophily occurs when the observed homophily effect is equal across all groups defined by individual characteristics, while differential homophily indicates that the homophily effect varies across different groups.

For example, one study on friendship formation among adolescent immigrants [66] found that the homophily effect of immigrant status differed significantly among immigrants with different generational statuses, with first-generation adolescent immigrants exhibiting the strongest homophily effect. Similarly, Sun [38] identified identity-driven differential homophily in Twitter health communication networks; stronger homophily effects were observed within patient and healthcare provider users, while a significant negative homophily effect was found within supporter users (i.e., who tended not to develop dialogue relationships with each other). In environmental communication research, Elgin [58] found that homophily played a positive role in establishing ties among advocates who adhered to scientific consensus on climate change, while skeptical actors demonstrated more diverse interactive behaviors with more cross-stance interactions.

In light of these findings on differential homophily effects, we propose our final research question:

**RQ3:**  In the formation of GMO risk information-sharing relationships, does the positional homophily effect exhibit differences amongst groups with different GMO stances?

To enhance the clarity of our theoretical framework, Table 1 summarizes the key theories and studies reviewed above and their specific relevance to our research questions.

## 3. Research Design

### 3.1. Data Collection

To investigate both endogenous and exogenous mechanisms driving the social sharing of GMO risk information, we collected GMO risk posts and their sharing records on Weibo.

Our dataset consists of 388,722 public reposts of 2444 original GMO risk-related Weibo posts. These original posts were selected through a systematic filtering process from a larger dataset of 356,227 GMO risk-related Weibo posts collected using a Python (version 3.9.0) crawler (detailed in [21,22]). To ensure meaningful analysis, we focused on the 117,499 original posts within this dataset and excluded posts with no reshares (77.28% or 90,809 posts). To address data sparsity in information cascades [62], we further filtered out messages with fewer than 50 reposts, resulting in 2670 original GMO risk posts for further analysis. We then gathered all reposting data to reconstruct complete information cascades. We successfully captured sharing records for 2444 original posts; theoretically, these posts encompassed 687,212 reposting interactions (averaging 281.18 reposts per post), though approximately 33.53% of reposts and their subsequent chains (230,389 entries) were unobtainable due to deletion, privacy settings, or platform limitations. Temporal analysis revealed that over 50% of reposts occurred within 24 h, and 75% within four days of the original post. The average reposting time was 49 days, with a maximum of 8.7 years (3177 days). Following Meng et al. [71]’s recommendation, we selected sharing relationships within two weeks of the original message date, resulting in our final dataset of 2444 original posts with 388,722 publicly visible reposting relationships involving 150,337 unique users.

Additionally, this study collected social profiles for all users involved in these information cascades, including follower count, following count, post count, and available demographic information, which will be detailed later.

### 3.2. Constructing the Sharing Network

Based on the sharing records, we constructed the GMO risk information-sharing network (hereafter “sharing network”).

Using the 2444 information cascades with 388,722 sharing relationships, we created an edgelist for building the sharing network. To ensure meaningful analysis of positional homophily, we refined this list to include only sharing relationships involving the 66,789 users whose GMO attitude scores could be calculated and identified (as described in the next section). Using the *igraph* package in R (version 4.1.0), we constructed a weighted directed sharing network, wherein when user A reposts content from user B, a directed edge is established from A to B, with the edge weight representing the frequency of these reposting interactions.

After extracting the largest connected component and removing self-loops, the final network comprised 56,432 nodes and 97,442 edges. We incorporated individual-level characteristics as node attributes, including GMO attitude score, account verification status, follower count (log-transformed), following count (log-transformed), post count (log-transformed), gender, and user activity level provided by Weibo.

The resulting sharing network exhibits several notable characteristics. It is extremely sparse (density < 0.0001), with a reciprocity of 0.058 and transitivity of 0.004. The network has an average path length of 5 and a diameter of 249. On average, each node connects to 3.453 other nodes (*SD* = 42.3, *Mdn* = 1, *Max* = 4148), indicating a highly skewed degree distribution typical of online social networks.

### 3.3. Detecting Users’ GMO Stance

To examine the positional homophily effect, we needed to identify each user’s attitude or stance toward GMOs. Prior studies typically rely on manual content analysis to identify user stance in expressed social media posts (e.g., ref. [67]). However, this approach has significant limitations since it can only analyze a small number of texts, and a single post may not accurately represent a user’s overall stance on GMOs. Other studies have employed behavioral data to infer user positions—for example, estimating Twitter users’ political ideology based on their following patterns [62,72,73]. While effective, this method requires user-following data that is not applicable to our study due to Weibo’s privacy settings.

Given the need to estimate GMO attitudes for a large number of users, we adopted a computational stance detection approach that combines manual coding with machine learning [74,75,76]. This approach first detects stances at the text level in users’ GMO-related posts and then aggregates these text-level attitudes to calculate each user’s overall GMO stance.

To implement this approach, we used a Python crawler to collect all Weibo posts (14,160,730 posts total, averaging 94.19 posts per user) published by the 150,337 users within an eight-day window—four days before and after their reposting behavior. We then filtered these posts for the keyword “Zhuanjiyin” (GMO in Chinese), yielding 760,706 GMO-related Weibo posts as the basis for text-level stance detection.

For training and validation, we randomly sampled 16,000 posts (2.1%) for manual coding. Four graduate students in communication study coded each post’s expressed GMO attitude into three categories: Support (1), Oppose (−1), or Neutral/Unclear (0). After multiple training rounds, the coders achieved a Krippendorff’s alpha of 0.83, indicating relatively strong inter-coder reliability. The labeled data was then split into a training set (12,800 posts) and a testing set (3200 posts).

We trained two deep learning models—FastText and Bert—on the training data and evaluated their performance on the testing set. The FastText classifier outperformed Bert with an overall accuracy of 0.814 (f1-scores of 0.870, 0.752, and 0.713 for the oppose, support, and neutral/unclear categories, respectively). Based on these results, we used the FastText model to predict attitudes in all 760,706 GMO-related Weibo posts.

After obtaining the predicted attitudinal categories for each post, we mapped these to their respective authors. To ensure reliable measurements, we excluded users with fewer than 5 GMO-related posts. Following Matuszewski and Szabó [65], we calculated GMO attitudinal scores for the remaining 66,789 users using the following formula:

(1) $$Score=\frac{N_{pro}+N_{netural}}{N_{pro}+2\times N_{netural}+N_{against}}\;,$$

in this formula, $N_{pro}$, $N_{netural}$, and $N_{against}$ represent the number of posts supporting, neutral toward, or opposing GMOs, respectively. The score ranges from 0 to 1: values closer to 1 indicate stronger support for GMOs, values closer to 0 indicate stronger opposition, and values around 0.5 suggest a neutral stance.

### 3.4. ERGM Specification and Measurement

To investigate the mechanisms influencing GMO risk information sharing, we employed Exponential Random Graph Models (ERGMs) [77,78]. ERGMs are particularly well-suited for this research as they function as logit models that use Markov chain Monte Carlo (MCMC) methods to assess the statistical probability of specific network configurations [78].

The primary advantage of ERGMs lies in their inferential approach based on null models. By comparing observed network variables against a large number of randomly generated networks, ERGMs allow us to explore the fundamental question of how ties form. Furthermore, ERGMs can integrate influencing factors from multiple levels (node, dyad, triad, and network-size attributes) to predict the probability of relationship formation between two nodes [40,42].

We converted the sharing network (an igraph object) into a network object compatible with ERGMs using the *intergraph* package in R. We then fitted the model using the *statnet* package, incorporating both endogenous network structural forces, positional homophily mechanism, and other exogenous nodal attributes to predict risk information-sharing behavior.

For endogenous mechanisms, this study focused primarily on the influence of reciprocity, preferential attachment, and triadic closure on sharing relationships. Following prior research [38,40,43], we incorporated the number of mutual dyads, Geometrically Weighted In-degree Distribution (GWID), and the Geometrically Weighted Edgewise Shared Partners (GWESP) to measure these three mechanisms, respectively. Additionally, we included the Geometrically Weighted Out-degree Distribution (GWOD), which is typically interpreted as a measure of “activity” or “activeness” in network research [38].

We also controlled for the number of asymmetric dyads at the network structure level to ensure model robustness. This control is particularly important given the relatively high proportion of users with opposing attitudes toward GMOs in the sharing network (*M* = 0.30, *SD* = 0.31, *Mdn* = 0.25, *Q3* = 0.50). Without this control, the estimated positional homophily effect could be biased. The inclusion of these control variables also helped prevent network degeneracy and achieve model convergence [38,78].

To measure the positional homophily effect, we calculated the absolute value of the difference in GMO attitude scores between dyads. A smaller value indicates greater similarity in GMO attitudes between two nodes. It is worth noting that while existing research often uses “homophily” functions (such as “nodematch”) to estimate homophily effects, the continuous nature of our GMO attitude score made the “absdiff” function more appropriate in this analysis.

For exogenous nodal attributes, we included gender, verified account status, number of Weibo posts (log-transformed), number of followers (log-transformed), number of followees (log-transformed), user activity level, and GMO attitude score. For the binary variables (gender and verified account status), we used the “nodefactor” function to examine their main effects; for continuous variables, we employed the “nodecov” function for effect estimation.

### 3.5. Shannon Entropy-Based Analysis

Prior to conducting the ERGM analysis, we employed Shannon entropy as an information-theoretic approach to quantify the uncertainty and dispersion in GMO attitudinal differences between users who share information versus those who do not. Shannon entropy provides a robust mathematical framework for measuring the degree of randomness or predictability in the distribution of positional differences, offering complementary insights to network-based ERGM analysis.

For each dyadic relationship (both sharing and non-sharing ties), we calculated the normalized Shannon entropy based on the absolute differences in GMO attitude scores. The entropy calculation was stratified across three key categories: all dyads, opponent dyads (where both users oppose GMOs), and non-opponent dyads (where users either support or are neutral toward GMOs). For non-sharing ties, we employed MCMC simulations to sample from the vast space of potential but unrealized connections, ensuring a balanced comparison with observed sharing ties.

This entropy-based analysis serves multiple purposes: (1) it provides an information-theoretic foundation for understanding the structure of attitude alignment in the network, (2) it offers preliminary evidence for the presence and strength of positional homophily effects that we later test through ERGMs, and (3) it helps identify potential differential homophily patterns across different user groups. The results of this analysis, detailed in the Results section, reveal systematic differences in entropy values between sharing and non-sharing ties, suggesting that information flow in the network is indeed influenced by attitudinal similarity patterns.

## 4. Results

### 4.1. Entropy in Sharing Network

**Figure 1:**
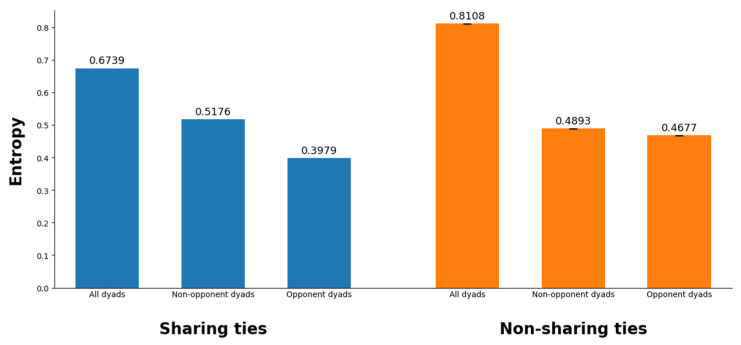
Normalized Shannon entropy in risk information sharing and non-sharing ties.

Shannon entropy values (see Figure 1) are computed for three dyadic categories: all dyads, opponent dyads, and non-opponent dyads, each further stratified by tie status (sharing versus non-sharing ties). Across all dyads, sharing ties exhibit lower entropy (H = 0.6739) compared to non-sharing ties (H = 0.8108), indicating that dyads engaged in risk information-sharing relationships display a more concentrated distribution of attitude differences, with individuals tending to share similar GMO attitudes. In contrast, non-sharing ties are characterized by greater variability in attitude differences, reflecting weaker alignment in GMO stance. The lower entropy in sharing ties to some extent supports the positional homophily hypothesis, suggesting that attitude similarity acts as a key driver of GMO risk information-sharing behavior.

Among opponent dyads, sharing ties demonstrate the lowest entropy (H = 0.3979), significantly lower than non-opponent dyads (H = 0.5176) in the same tie category. This stark contrast underscores the heightened role of positional homophily within groups opposing GMOs. The extreme concentration of attitude differences among sharing opponent dyads suggests that individuals who oppose GMOs are highly selective in sharing risk information, primarily interacting with others who share their skepticism. Notably, even in non-sharing ties, opponent dyads exhibit marginally lower entropy (H = 0.4677) than non-opponent dyads (H = 0.4893), hinting at residual positional homophily-driven clustering among opponents, albeit without active information exchange. It is also noteworthy that non-opponent dyads display higher entropy in both sharing (H = 0.5176) and non-sharing (H = 0.4893) ties compared to the opponent group. This indicates greater variability in attitudinal similarities among GMO supporters or neutrals, even when information-sharing occurs.

In summary, the entropy patterns shown in Figure 1 highlight three critical aspects. First, the low entropy in sharing opponent dyads signals the formation of tightly aligned “echo chambers,” where GMO risk information propagates within homogenous clusters of skeptics, potentially reinforcing pre-existing beliefs. Second, the high entropy in non-sharing ties suggests that cross-cutting interactions—where individuals with divergent GMO attitudes could exchange information—are relatively rare. This absence of bridging ties may exacerbate polarization, as opposing groups remain isolated in their communication networks. Finally, the relatively higher entropy in non-opponent dyads compared to their opponent counterpart suggests a differential homophily mechanism.

Notwithstanding, the above-mentioned insights concerning differential positional homophily derived from this entropy-based analysis are possibly subject to confounding effects from network structural factors/constraints that may influence information-sharing behavior. The entropy approach alone cannot account for these endogenous network forces that might affect the observed patterns. Therefore, in what follows, we will systematically investigate the effects of positional homophily and differential homophily by employing ERGM with endogenous network structural mechanisms being controlled for, allowing us to examine both the endogenous mechanisms driving behavioral cascades and the exogenous positional homophily effects simultaneously.

### 4.2. Endogenous Mechanisms and Information Sharing

RQ1 asks the influence of endogenous mechanisms on GMO risk information sharing. Table 2 presents the ERGM results, revealing several significant patterns in the network structure. As shown in the full network, the parameter estimate for the number of mutual dyads (mutuality) is significantly positive (*b* = 6.66, *p* < 0.001), indicating that the sharing network exhibits greater reciprocity compared to a random network. This suggests that users’ information-sharing behavior follows strong reciprocity norms. Interestingly, the coefficient for asymmetric dyads was also significantly positive (*b* = 0.65, *p* < 0.001), suggesting that while reciprocity plays a dominant role in tie formation, some sharing behaviors still exhibit non-reciprocity.

The positive coefficient for GWID (*b* = 3.71, *p* < 0.001), which measures the overall popularity effect of nodes at the network level, indicates high centralization based on node in-degree. This reveals that a small number of influential users dominate the sharing behavior of many users, demonstrating the significant influence of preferential attachment in GMO risk information sharing. Correspondingly, GWOD, which measures the overall activity of nodes, shows a negative coefficient (*b* = −5.55, *p* < 0.001), implying that most users have relatively similar levels of activity in sharing GMO risk-related Weibo posts. This finding indirectly confirms the existence of the preferential attachment mechanism.

Additionally, the model shows that GWESP positively influences the probability of sharing relationship formation (*b* = 2.30, *p* < 0.001), meaning that the probability of connecting and completing transitive triangles is 9.97 times higher than in a random network (exp(2.30)). This result suggests that the triadic closure mechanism plays an important role in users’ GMO risk information-sharing decisions and behaviors.

### 4.3. Positional Homophily and Differential Effects

RQ2 investigates the impact of positional homophily on GMO risk information sharing. As shown in the first column of Table 2, users’ GMO attitudinal score positively influences the formation of risk information-sharing relationships (*b* = 0.38, *p* < 0.001). For every one-unit increase in a user’s GMO attitude score, the odds of that user forming a sharing relationship with others increase by 46.23% (exp(0.38) − 1). This suggests that users with more positive attitudes towards GMOs are more likely to participate in sharing GMO risk information.

Regarding the positional homophily effect, the ERGM results show that the coefficient for the absolute difference in dyadic GMO attitudinal scores is significantly negative (*b* = −2.07, *p* < 0.001). When the absolute difference in GMO attitude scores between two users is 1 (one user extremely opposes GMOs, and the other extremely supports them), the probability of them forming a sharing relationship decreases by 87.38% (1 − exp(−2.07)). In other words, users are more likely to share information with others who hold similar attitudes toward GMOs, confirming the presence of a strong positional homophily mechanism in GMO risk information sharing.

To further explore whether the positional homophily effect differs between groups with different GMO stances (RQ3), we followed Matuszewski and Szabó’s [65] approach. Users were divided into three groups—GMO opponents, neutrals, and supporters—based on their attitudinal scores, with cut-off points at 0.4 and 0.6. The neutral and supporter groups were further merged to create two sub-networks: the opponent and the non-opponent sharing network.

The second and third columns of Table 2 present the ERGM results for these sub-networks. The estimates for positional homophily effect in both sub-networks are consistent with the overall sharing network, both being significantly negative. This indicates that both GMO opponents (*b* = −2.63, *p* < 0.001) and non-opponents (*b* = −1.03, *p* < 0.001) tend to share posts published by users whose attitude stance is more consistent with their own.

Notably, we found a significant difference in the strength of the positional homophily effect between these groups: the coefficient for opponents is nearly 2.5 times that of non-opponents. This finding suggests that the positional homophily effect is more pronounced among users who hold opposing attitudes towards GMOs compared to those who hold neutral or positive views. This corroborates the concept of differential homophily [38,70] in the context of GMO risk communication, suggesting that different social groups may exhibit varying degrees of homophilic tendencies in their information-sharing behaviors.

## 5. Robustness Check

The positional homophily effect identified in our analysis warrants careful interpretation. Traditional approaches to identifying homophily often employ a simplistic framework: when individuals with similar attributes show a greater tendency to form connections, researchers typically attribute this to homophily. However, this approach may overlook important confounding mechanisms that influence the estimation of homophily effects.

Among these potential confounding factors, the “avoidance mechanism” [42,58] deserves particular attention. This mechanism suggests that the higher frequency of within-group interactions may result not from users’ preference for similar others (homophily) but rather from their deliberate avoidance of interactions with users holding different viewpoints. This distinction is crucial for accurately understanding network formation dynamics in polarized online discussions.

Previous research illustrates the importance of controlling for such mechanisms. For instance, Schmid-Petri et al. [42] found that when controlling for cross-camp avoidance behaviors in climate change discussions, homophily effects remained significant among climate change supporters but disappeared among climate change skeptics. This highlights how different mechanisms may operate simultaneously but with varying strengths across different user groups.

To address this methodological concern, we followed Schmid-Petri et al.’s [42] approach by employing the “nodemix” function in our ERGM model to control for “opponent-non-opponent” disassortative sharing relationships. The results (presented in the fourth column of Table 2) reveal two important findings. First, even after controlling for disassortativity relationships, positional homophily remains a significant mechanism driving sharing relationship formation. This confirms the robustness of our earlier findings regarding homophily effects.

Second, and perhaps more intriguingly, we found that disassortative relationships (sharing between opponents and non-opponents) occur with significantly higher probability than would be expected in a random network. Specifically, 20.21% of sharing relationships were observed between users with opposing GMO attitudes, and the probability of such cross-cutting interactions was 1.78 times higher (exp(0.58)) than in a random network.

These results contribute important nuance to ongoing debates about echo chambers and cross-cutting exposure in social media discussions of controversial socio-scientific topics. They suggest that while users do exhibit homophilic tendencies in their sharing behaviors, the online public sphere remains somewhat permeable to cross-stance interactions.

## 6. Discussion

This study examines the behavioral dynamics of GMO risk information diffusion on social media, with a particular focus on sharing behaviors and the underlying mechanisms that drive these collective actions. By drawing upon social contagion theory and the MTML analytical framework [36,37,38], we integrate both endogenous network structural mechanisms and positional homophily to understand how GMO risk information spreads through social networks. This approach represents a significant departure from traditional risk communication research that has primarily focused on message content [11,12,13] or individual cognitive processes [26,27,35]. Our study effectively recenters the “social” dimension in the “social construction of risk” [79,80,81].

The findings demonstrate that both endogenous network structures and homophily mechanisms significantly influence risk information-sharing behavior among Weibo users discussing GMO-related issues. These results align with key findings from other communication domains, including political, environmental, and health communication [37,38,40,42].

### 6.1. Endogenous Network Structural Mechanisms

Regarding RQ1 on endogenous network structural mechanisms, our analysis reveals strong reciprocity norms among users in the sharing network, confirming the significant influence of reciprocity on GMO risk information-sharing behavior. The study also demonstrates high transitivity in sharing behavior, validating the positive role of triadic closure in forming sharing relationships. This transitivity promotes the development of cohesive risk information-sharing networks [38], facilitating the widespread and effective dissemination of GMO risk messages.

Additionally, we confirm the existence of a preferential attachment mechanism, manifested in the higher centralization of the sharing network’s overall in-degree (GWID) compared to random networks. Within the sharing network, a small number of highly influential users form sharing relationships with the majority of peripheral users, while information sharing among these peripheral users occurs less frequently. This preferential attachment pattern in GMO risk information diffusion resembles the unequal discursive order in the online co-production of GMO risk issues identified by Cheng [21], both reflecting the power-law distribution commonly observed in social media discussions of GMO risk issues [15,49]. Such patterns can potentially lead to a public sphere dominated by a small number of prominent voices.

### 6.2. Positional and Differential Homophily: Insights from ERGM and Entropy

Homophily, as a fundamental organizing principle in human society [52], constitutes an important mechanism in the formation of GMO risk information-sharing relationships on social media. Our ERGM results confirm the impact of positional homophily on information sharing (RQ2) and further reveal the phenomenon of differential homophily (RQ3) in users’ sharing behaviors [38,70].

The entropy-based analyses complement and reaffirm the ERGM results by quantifying the degree of attitude alignment underlying sharing and non-sharing ties. The results indicate that positional homophily is a stronger predictor of information sharing among GMO opponents, while non-opponent groups exhibit more flexibility in their sharing and communication patterns. This pattern demonstrates that attitudinal similarity plays a pronounced role in shaping the sharing behaviors of certain subgroups.

To contextualize these findings, we compare them with previous network studies examining risk information sharing dynamics. Komori et al. [13] investigated the interplay between network structure and risk information sharing on Twitter, finding that both centrality and mutuality scores were negatively related to risk information retweeting; importantly, they identified a significant interaction between mutuality and emotional risk narrative: for users with high mutuality (many reciprocal connections), the level of dread expressed in risk posts elevated their retweeting rate. This suggests that high-mutuality users may share risk information primarily for relationship maintenance and emotional expression rather than information exchange, while low-mutuality users appeared motivated primarily by information exchange purposes. Our entropy-based results, which highlight stronger homophily effects among GMO opponents, to some extent resonate with Komori et al.’s [13] observation that network characteristics moderate the mechanisms underlying risk information sharing. While our study focuses on attitudinal alignment rather than emotional content and responses, the observed subgroup differences in homophily strength suggest similar interaction effects: certain user groups (e.g., GMO opponents) may be more motivated by affective or identity-related considerations, leading to more selective and homophilous sharing patterns.

Despite the strong homophily effects observed, our robustness checks reveal an intriguing finding: there exists a meaningful degree of cross-attitude sharing behavior between users with different attitudinal stances. After controlling for the avoidance mechanism, we found that the probability of disassortative relationships (sharing between opponents and non-opponents) appearing in the network is significantly higher than would be expected in a random network. This suggests that while homophily is indeed a dominant mechanism, social media platforms such as Weibo still function as public forums for GMO risk discussions where cross-cutting discourse occurs.

This finding aligns with Wang and Song’s [67] research and contributes to the ongoing debate about whether social media platforms facilitate echo chambers or cross-cutting discourse. Our results indicate a more nuanced reality: while users do exhibit homophilic tendencies in their sharing behavior, particularly among GMO opponents, there remains significant cross-stance interaction that prevents the complete fragmentation of the discourse.

A particularly noteworthy finding is the differential positional homophily we observed: compared to users with neutral or positive attitudes towards GMOs, users who oppose GMOs demonstrate stronger homophilic tendencies in their sharing behaviors. This difference may stem from the heterogeneity in information processing mechanisms through which different groups form their GMO attitudes. Previous research has identified two distinct mechanisms: cognition-based and value predisposition-based. The former primarily forms attitudes based on judgments of scientific facts, while the latter is more susceptible to heuristic factors such as motivated reasoning [82,83].

These different attitudinal formation mechanisms affect how various publics understand and judge scientific knowledge, leading to varying degrees of what Malka, Krosnick, and Langer [84] call the biasing effect of overconfidence. For instance, research on public attitudes towards GMOs has found that individuals with the most extreme anti-GMO stances often possess limited objective scientific knowledge about GMOs, yet paradoxically believe they are highly knowledgeable (reporting high subjective knowledge). This cognitive bias can inhibit the acquisition of scientific knowledge among anti-GMO individuals, causing them to increasingly rely on non-cognitive factors, such as values and affect, to form their attitudes [85,86].

Consequently, when users with opposing stances encounter GMO risk-related information on social media, they are more likely to share information that confirms their prior attitudes due to confirmation bias. An experimental study conducted by Jang [87] supports this inference, showing that individuals who believe they have sufficient scientific knowledge and strong religious beliefs tend to engage with GMO information that aligns with their pre-existing attitudes rather than information that challenges them. In contrast, GMO supporters tend to rely more on cognition-based mechanisms when processing risk information, carefully evaluating the accuracy of the information provided, which may make them more tolerant of counter-attitudinal information.

### 6.3. Theoretical and Practical Implications

This study makes several theoretical contributions to our understanding of information diffusion in digital environments. First, by integrating social contagion theory with the MTML framework, we demonstrate how multiple mechanisms—reciprocity, transitivity, preferential attachment, and homophily—simultaneously shape risk information sharing. This multi-theoretical approach provides a more comprehensive model for understanding the complex dynamics of information diffusion than single-mechanism explanations.

Second, our findings on differential homophily—whereby GMO opponents exhibit stronger homophilic tendencies than non-opponents—add a new layer to contagion theory, suggesting that the strength and direction of behavioral contagion effects can vary systematically across attitudinal subgroups. This insight underscores the importance of considering group-specific mechanisms and a segmentation approach in models of information diffusion, moving beyond the assumption of uniform contagion processes.

Third, our study contributes to the theoretical understanding of echo chambers by revealing a more nuanced picture than the binary “echo chambers vs. cross-cutting exposure” debate prevalent in the literature [21]. By quantifying both homophilic tendencies and cross-attitudinal sharing, we demonstrate that these phenomena coexist within the same communication network, suggesting that theoretical models need to account for this complexity rather than treating them as mutually exclusive outcomes.

Our findings also offer several practical implications for stakeholders involved in risk communication. For science communicators, the pronounced homophily among GMO opponents suggests the need for tailored strategies that address this group’s specific information processing patterns. Interventions designed to foster critical engagement and reduce confirmation bias may be particularly effective for this audience. The observed preferential attachment pattern indicates that highly influential users play disproportionate roles in information diffusion, suggesting that engaging these key influencers could facilitate more effective cross-attitudinal communication.

For policymakers, our results highlight the importance of understanding social dynamics when developing policies related to controversial scientific topics. The coexistence of homophilic tendencies and cross-cutting discourse suggests that policy approaches should neither assume complete polarization nor expect uniform information reception across different public segments. Policies that encourage dialogue across attitudinal divides while acknowledging legitimate concerns from all perspectives may be most effective.

Finally, social media platform designers could leverage these insights to develop features that maintain exposure to diverse viewpoints while respecting user preferences. For instance, recommendation algorithms could be adjusted to ensure some degree of viewpoint diversity in users’ feeds, thereby mitigating echo chamber effects without completely disrupting user engagement.

### 6.4. Limitations and Future Directions

Several limitations of this study should be acknowledged. First, our analysis focuses exclusively on Weibo, a Chinese social media platform, which may limit the generalizability of our findings to other cultural contexts and social media platforms. Second, our inclusion threshold of 50 reposts may have excluded potentially important diffusion patterns in less viral content. Third, our study focuses exclusively on reposted posts, which may limit our understanding of the complete information-sharing landscape. A comparative analysis between reposted and non-reposted risk messages could provide insights into why certain pieces of GMO risk information are shared, while others are not, potentially uncovering content-specific characteristics or contextual factors that determine information virality beyond the network mechanisms and homophily effects identified in this study.

Several promising directions could significantly advance our understanding of risk information diffusion in digital environments. A particularly important avenue involves examining the role of misinformation in the network dynamics we observed. The contemporary digital information landscape is increasingly characterized by the proliferation of misinformation [88], which poses significant challenges for effective risk communication. Research has demonstrated that false information spreads faster and more widely than accurate information on social networks, with misinformation reaching more people and penetrating deeper into social networks than the truth [89]. This phenomenon is particularly concerning in the context of scientific and risk-related topics like GMOs, where misinformation can lead to harmful public health outcomes and undermine evidence-based decision-making [90]. In this regard, future studies could investigate whether the homophilic tendencies we observed facilitate the spread of misinformation within like-minded communities, as users may be less likely to critically evaluate information that confirms their existing beliefs. Specifically, researchers could examine whether homophily effects are stronger for false information than for accurate information, and whether the differential homophily we observed among GMO opponents makes them more susceptible to misinformation.

Another critical direction involves examining how platform governance structures influence the network formation patterns and information diffusion mechanisms identified in this study. Platform companies implement different content control mechanisms, ranging from automated detection systems to human moderation, which significantly influence information visibility and user engagement patterns [91]. These governance approaches vary considerably between democratic and less- or non-democratic contexts, with implications for the diversity and accuracy of information available to users [92,93]. Considering this, cross-platform comparative studies could provide valuable insights into how different regulatory frameworks and content moderation policies affect the formation and strength of homophilic sharing networks in risk communication contexts. For instance, researchers could compare information diffusion patterns on platforms with strict content moderation versus platforms with more permissive policies. Such comparisons could reveal whether platform governance structures amplify or mitigate the homophily effects we observed, and how they influence the balance between echo chamber formation and cross-cutting discourse.

## 7. Conclusions

In conclusion, this study provides valuable insights into the behavioral mechanisms underlying GMO risk information diffusion on social media, highlighting the complex interplay between network structures and homophily in shaping sharing behaviors. By demonstrating both the presence of homophilic tendencies and meaningful cross-cutting discourse, our findings contribute to a more nuanced understanding of how risk information spreads in digital and polarized environments and how different attitudinal groups engage with such information. These insights have important implications for risk communication strategies and for understanding the role of social media in shaping public discourse around controversial socio-scientific risk topics.

## Figures and Tables

**Table 1:** Summary of key studies and their relevance to RQs.

Research Question (RQ)	Key Studies	Core Concepts	Relevance to RQ
**RQ1**	[42,46]	**Preferential attachment**: new connections favor nodes with higher degrees (“rich get richer”)	Explains how popular and influential users attract more sharing relationships in GMO networks
	[39,40,50]	**Reciprocity**: humans seek symmetry in relationships **Triadic closure**: “friends of friends become friends”	Explains mutual sharing behaviors and clustering in GMO information networks
**RQ2**	[51,52,53]	**Homophily**: similarity breeds connection	Explains tendency for users with similar GMO attitudes to share information
	[42,60]	**Positional homophily**: relationship formation based on shared attitudinal positions	Specifically addresses attitude-based connections in GMO risk communication
**RQ3**	[38,58,70]	**Differential homophily**: homophily effects vary across different groups	Explains potential differences in sharing patterns between GMO supporters and opponents

**Table 2:** Comparison of ERGM results.

	Full Network	Opponent Network	Non-Opponent Network	Full Network -Avoidance
**Edges**	−13.68 ***	−11.90 ***	−20.95 ***	−13.03 ***
**Endogenous mechanisms**				
Mutual dyads	6.66 ***	5.11 ***	4.93 ***	4.28 ***
GWID	3.71 ***	1.82 ***	3.61 ***	3.04 ***
GWESP	2.30 ***	2.34 ***	1.41 ***	0.70 ***
**Endogenous mechanisms-controls**				
GWOD	−5.55 ***	−4.81 ***	−4.58 ***	−6.43 ***
Asymmetric dyads	0.65 ***			0.74 ***
**Positional homophily mechanism**				
Absolute value of the difference in GMO attitudinal scores (absdiff)	−2.07 ***	−2.63 ***	−1.03 ***	−2.68 ***
**Avoidance Mechanism**				0.58 ***
**Exogenous nodal attributes**				
GMO attitudinal score	0.38 ***	1.43 ***	1.08 ***	0.44 ***
Male	−0.04 ***	−0.04 ***	−0.32 ***	−0.01
Weibo posts (log)	−0.05 ***	−0.25 ***	0.62 ***	−0.03 ***
Followers (log)	0.77 ***	0.90 ***	0.96 ***	0.61 ***
Followees (log)	−0.08 ***	−0.09 ***	−0.09 ***	0.07 ***
User activity level	−0.01 ***	−0.01 ***	−0.02 ***	−0.00 ***
Verified account	−0.74 ***	−0.84 ***	−0.23 ***	−0.80 ***
**AIC**	1,743,069.30	1,094,668.67	225,495.57	1,747,697.86
**BIC**	1,743,347.64	1,094,914.96	225,728.45	1,747,996.09
**Log Likelihood**	−871,520.65	−547,321.34	−112,734.79	−873,833.93

*** *p* < 0.001.

## Data Availability

The datasets generated or analyzed during the current study are not publicly available as they form part of the author’s ongoing research. They are available from the corresponding author on reasonable request.

## Abbreviations

The following abbreviations are used in this manuscript:

GMO	genetically modified organism
ERGM	Exponential Random Graph Model
MTML	Multi-Theoretical Multilevel model
